# Selecting an optimal approach to reduce energy crises under interval-valued intuitionistic fuzzy environment

**DOI:** 10.1038/s41598-024-57164-1

**Published:** 2024-04-15

**Authors:** Dilshad Alghazzawi, Hanan Alolaiyan, Humaira Ashfaq, Umer Shuaib, Hamiden Abd El-Wahed Khalifa, Heba Ghareeb Gomaa, Qin Xin

**Affiliations:** 1 Department of Mathematics, College of Science & Arts, King Abdul Aziz University, Rabigh, Saudi Arabia; 2 Department of Mathematics, College of Science, King Saud University, Riyadh, Saudi Arabia; 3 Department of Mathematics, Government College University, Faisalabad, 38000 Pakistan; 4 Department of Mathematics, College of Science, Qassim University, Buraydah, 51452 Saudi Arabia; 5 Department of Operations and Management Research, Faculty of Graduate Studies for Statistical Research, Cairo University, Giza, 12613 Egypt; 6 Institute for Management Information Systems, Suez, Egypt; 7 Faculty of Science and Technology, University of the Faroe Islands, Vestara Bryggja 15, FO 100 Torshavn, Faroe Islands Denmark

**Keywords:** Interval valued intuitionistic fuzzy set, Renewable energy source, Similarity measure, Decision making, Optimization, Engineering, Mathematics and computing

## Abstract

The concept of interval-valued intuitionistic fuzzy sets is intellectually stimulating and holds significant utility in the representation and analysis of real-world problems. The development of similarity measures within the class of interval-valued intuitionistic fuzzy sets possesses significant importance across various academic disciplines, particularly in the fields of decision-making and pattern recognition. The utilization of similarity measures is of utmost importance in the decision-making process when implementing interval-valued intuitionistic fuzzy sets. This is due to its inherent capability to quantitatively assess the level of resemblance or similarity between two interval-valued intuitionistic fuzzy sets. In this article, the drawbacks of the existing similarity measures in the context of an interval-valued intuitionistic fuzzy environment are addressed, and a novel similarity measure is presented. Many fundamental properties of this new interval-valued intuitionistic fuzzy similarity measure are also established, and the effectiveness of this similarity measure is illustrated by presenting a useful example. Moreover, a comparison is given to demonstrate the validity of the newly proposed similarity measure within the existing knowledge of similarity measures in the interval-valued intuitionistic fuzzy environment. In addition, an algorithm is designed to solve multi-criteria decision making problems by means of the proposed measure in the interval-valued intuitionistic fuzzy setting. Furthermore, this newly defined similarity measure is successfully applied to select an optimal renewable energy source to reduce energy crises. Finally, we conduct a comparative study to showcase the authenticity of the recently defined technique within the existing knowledge of similarity measures in the interval-valued intuitionistic fuzzy environment.

## Introduction

The process of decision making entails the selection of a particular plan of action from a range of available alternatives. This is the process of choosing the most optimal alternative from a range of available options. Multiple criteria decision making (MCDM) is a way to figure out the best way to deal with problems that are related to certain criteria that come up in everyday life. Given the complex nature of the societal context and the limited availability of precise information, decisions are commonly arrived at through the collective efforts of a group of specialists, as opposed to being made by individual entities. MCDM serves to decide the best option among several options. Recent decades have seen the rapid development of multi-criteria fuzzy decision making due to its wide application.

Zadeh^1^ developed the notion of a fuzzy set (FS). It appears that the human brain processes verbal adjectives such as "brilliant" and "more brilliant," "long," "very long," "tall," and "very tall," at a faster rate than numerical figures. The capacity of human intelligence encompasses the ability to reduce intricate information to functional simplifications that closely resemble the original. Each element of the universal set is assigned a closed unit interval value by a membership function in an FS in order to ascertain its membership in the set under consideration. Humans frequently fail to add a degree of non-membership to 1 when stating the degree of membership of an element in an FS. The fact that logical negation does not always match verbal negation highlights a psychological truth. Atanassov^2^ suggested intuitionistic fuzzy sets (IFSs), which handle uncertainty better than fuzzy sets. This IFS generalization adds complexity to degrees of membership and non-membership, increasing information and semantic representation. The notion of interval-valued fuzzy sets (IVFS) was initially introduced by Zadeh^3^s in 1975. Nevertheless, numerous researchers contend that offering an expert opinion as a singular numerical value is infeasible, advocating instead for the presentation of a spectrum of credible values. In 1993, Gau and Buehrer pioneered the concept of vague sets^4^. Later on, Bustine and Burillo^5^ discovered in 1996 that the theories of vague sets and intuitionistic fuzzy sets are similar.

Hong and Kim^6^ presented an innovative IFS measure. Liang and Shi^7^ examined the use of three innovative similarity metrics for IFSs that they had introduced in 2003. In^8^, Szmidt and Kacprzyk developed a novel similarity metric for IFSs based on geometric principles. Ye^9^ devised a cosine similarity metric for IFSs. In 2014, a biparametric measure of similarity for IFSs was developed in^10^. In^11^, Ngan et al. introduced an IFS similarity metric in 2018 that is predicated on the cross-evaluation factor's maximum value. In 2019, Dhivya and Sridevi^12^ introduced an innovative similarity metric for IFS that utilizes the fuzzy number associated with the right-angled triangle as opposed to the centroid. In^13^, Jiang et al. established a similarity measure for IFS on the basis of a distance metric. Dengfeng and Chutting^14^ established the axiomatic definition of a similarity measure for IFSs, thereby introducing the measure. In^15^, the authors devised a similarity metric for the vague set. A similarity measure for IFS using Hausdorff distance was put forward in^16^. Wang and Xin^17^ introduced an innovative IFS distance metric. The distance measure or connection between membership and non-membership functions was utilized to develop an IFS similarity measure by Songs et al.^18^. Garg and Rani^19^ introduced an IFS similarity metric in 2021 that utilizes a transformed right triangle as its foundation. In^20^, Gohain et al. defined two IFS similarity measurements. In^21^, another innovative similarity measure for IFS was developed. Chen and Liu^22^ introduced a similarity metric for IF values pertaining to IFS. Kumar and Kumar^23^ introduced a novel similarity metric for IFS and implemented it in the context of pattern recognition and clustering. Utilizing intuitionistic fuzzy pairings, Ejegwa and Agbetayo^24^ designed and implemented the similarity-distance decision-making approach in 2023. Saqlain et al.^25^ defined distance and similarity metrics utilizing the IF hypersoft set.

Because of the binherent uncertainty and intricacy of physical problems, problems involving criteria values expressed as intervals have emerged as a compelling area of research within the domain of MCDM. IVIFSs, which are expansions of IFSs and IVFSs, were introduced by Atanassov and Gargov^26^. They feature a subinterval of [0,1], which signifies the extent of membership, non-membership, and hesitation, respectively.

In the scope of IVIFS, Xu and Chen^27^ established crucial IFS similarity measures. Wei et al.^28^ proposed a measure of similarity for IVIFS and used it to address pattern recognition and medical diagnostics problems. Singh^29^ developed and implemented a new cosine similarity measure for IVIFS in pattern recognition. In the context of the IVIFS environment, Khalaf^30^ suggested a novel technique for medical diagnosis. In^31^, the authors proposed a new similarity measure for IVIFSs with the help of modified fuzzy numbers. To overcome MCDM challenges in the IVIF environment, Luo and Liang^32^ created a new similarity measure. Ye and Du^33^ proposed an interval-valued neutrosophic set similarity measure. Based on the non-hasty score function, Jeevaraj^34^ established a new similarity measure for the class of IVIF numbers in 2020. Verma and Merigo^35^ proposed a cosine similarity measure for IVIFS in 2020, based on the concept of weighted reduced intuitionistic fuzzy sets. Tiwari and Gupta^36^ created an interval-valued intuitionistic fuzzy soft set distance, similarity, and entropy measure. In^37^ the authors created an IVIFS cosine similarity measure. A cosine similarity measure to tackle the MCDM problem with IVIF knowledge is developed in^38^. Nayagam et al.^39^ proposed a similarity measure based on the accuracy score of traditional trapezoidal-valued intuitionistic fuzzy sets. Jia et al.^40^ use the TOPSIS method for the evaluation of LCD in the world’s 47 countries by combining the FAHA.

The concept of IFS is unable to deal with difficulties that involve a range of possible values, just as IVFS is unable to deal with problems that entail non-membership; therefore, IVIFS is a more accurate approach for dealing with scenarios like these. They allow membership and non-membership degrees to be defined as interval values, which improves uncertainty assessment and information representation. They also provide consistent interval value descriptions. These sets are crucial because they ease information collection, aid decision-making, and handle uncertainty in several fields.

IVIFS depends heavily on similarity measures since they quantify the degree to which two entities are alike or different from each other. Similarity measures are an integral part of IVIFS because they make it feasible to compare, rank, and categorize the system's data. They facilitate easier decision-making in a variety of fields, including pattern recognition, information retrieval, and recommendation systems, among others. This research paper presents a mathematical approach to tackle MCDM challenges. We accomplish this by introducing novel similarity measures within the IVIF setting. In addition, we apply the recently established approach to address the MCDM problem in order to showcase the effectiveness of IVIFS using similarity measures.

Every aspect of life and world activity requires energy. Many production and consumption activities require energy, which drives economic growth. We need immediate, cheap solutions for global energy shortages. Due to their environmental effect and high import costs, conventional energy sources cannot create clean energy. Energy shortages have harmed the economy, businesses, and individuals. Pakistan needs cheap, clean energy now due to its weak infrastructure. Today's world worries about fossil fuel depletion and rising energy prices. Renewable energy and technologies may help developing nations overcome energy issues. Pakistan's energy crisis may be solved faster and cheapest by switching to renewable energy. A "renewable energy source" regenerates spontaneously and eternally. Building a sustainable energy infrastructure and decreasing emissions requires these renewable resources. They preserve the environment by preventing climate change, acid rain, and fossil fuel pollution. Renewable energy systems are cheaper to operate and maintain than fossil fuel power plants. As technology develops and economies of scale are reached, renewable energy prices will fall, approaching traditional energy sources. High usage of renewable energy sources minimizes air pollution and emissions, lessening the risk of respiratory illnesses, cardiovascular diseases, and early death. Thus, renewable energy benefits finance, society, and technology. Renewable energy sources can meet rising energy needs and help build a sustainable energy future. In this paper, we propose a mechanism to select an optimal renewable energy source to reduce energy shortages using the proposed IVIFS similarity measure.

The remaining part of this work is briefly summarized as follows: In Sect. 2, we go through a few common definitions related to IVIFS. In Sect. 3, we discuss the shortcomings of various existing similarity measures defined on IVIFS. In Sect. 4, we introduce a new similarity measure on IVIFS and set up a comparison to demonstrate how useful the proposed similarity measure is. In Sect. 5, we present a step-by-step mathematical mechanism to solve MCDM problems by using the newly defined similarity measure in the IVIFS setting. Moreover, we apply this novel technique to choose an optimal renewable energy source to reduce energy crises in the IVIFS environment. Furthermore, we undertake a comparative analysis to demonstrate the validity of the newly proposed technique compared to the existing knowledge of similarity measures in the IVIFS environment. Additionally, we address the paper's findings in the concluding section.

## Preliminaries

In this section, we will provide a concise overview of the fundamental principles that are essential for subsequent analysis.

### Definition 2.1

^3^ Consider $$U$$ as the universal set. An IFS $${\rm K}$$ of $$U$$ is described as follows: $${\rm K} = \left\{ {\left\langle {u, \gamma_{\rm K} \left( u \right), \sigma_{\rm K} \left( u \right)} \right\rangle \left| {u \in U} \right. } \right\}$$$$,$$ where $$\gamma_{\rm K} :U \to \left[ {0 , 1} \right]$$ and $${\sigma }_{\rm K}:U\to \left[0 , 1\right]$$ are membership and non-membership functions, respectively. These functions are bounded by the following condition: $$0 \le {\gamma }_{\rm K}\left(u\right)+{\sigma }_{\rm K}\left(u\right)\le 1$$. Furthermore, the degree of hesitation for IFS $${\rm K}$$ is described as: $${\pi }_{\rm K}\left(u\right)=1-{\gamma }_{\rm K}\left(u\right)+{\sigma }_{\rm K}\left(u\right)$$.

### Definition 2.2

^6^ Let $U$ be the universal set and $D(I)$ be the collection of all subintervals of the closed unit interval $I$. An IVIFS ${\rm K}$ on $U$ is defined as follows: ${\rm K} = \left\{ {\left\langle {u, \gamma_{\rm K} \left( u \right), \sigma_{\rm K} \left( u \right)} \right\rangle \left| {u \in U} \right. } \right\},$ where $\gamma_{\rm K} \left( u \right) = \left[ { \gamma_{\rm K}^{ - } \left( u \right), \gamma_{\rm K}^{ + } \left( u \right)} \right]$ and ${\sigma }_{\rm K}\left(u\right)=\left[ {\sigma }_{\rm K}^{-}\left(u\right), {\sigma }_{\rm K}^{+}\left(u\right) \right].$ Moreover, ${\gamma }_{\rm K}:U\to D(I)$ and $\sigma_{\rm K} :U \to D\left( I \right)$ represent the membership and non-membership functions, respectively, subject to the conditions $0 \le {\gamma }_{\rm K}^{-}\left(u\right)+{\gamma }_{\rm K}^{+}\left(u\right)\le 1$, and $0 \le {\sigma }_{\rm K}^{-}\left(u\right)+{\sigma }_{\rm K}^{+}\left(u\right)\le 1$. Furthermore, we can compute the hesitance degree, ${\pi }_{\rm K}\left(u\right)=\left[ 1- {\gamma }_{\rm K}^{+}\left(u\right)- {\sigma }_{\rm K}^{+}\left(u\right), 1- {\gamma }_{\rm K}^{-}\left(u\right)- {\sigma }_{\rm K}^{-}\left(u\right)\right].$

### Definition 2.3

^32^ Any two IVIFS ${\rm K}$ and $\Lambda$ of a universe $U$ admit the following relation:

- $$\mathrm{A }\subseteq \mathrm{ B }\Leftrightarrow {\gamma }_{\rm K}^{-} \left({\text{u}}\right)\le {\gamma }_{\Lambda }^{-} \left({\text{u}}\right), {\gamma }_{\rm K}^{+} \left({\text{u}}\right)\le {\gamma }_{\Lambda }^{+} \left({\text{u}}\right), {\sigma }_{\rm K}^{-} \left({\text{u}}\right)\ge {\sigma }_{\Lambda }^{-} \left({\text{u}}\right)\mathrm{ and}{ \sigma }_{\rm K}^{+}\left({\text{u}}\right)\ge {\sigma }_{\Lambda }^{+} \left({\text{u}}\right)$$
- $${\text{A}}=\mathrm{ B }\Leftrightarrow {\gamma }_{\rm K}^{-} \left({\text{u}}\right)={\gamma }_{\Lambda }^{-} \left({\text{u}}\right), {\gamma }_{\rm K}^{+} \left({\text{u}}\right)={\gamma }_{\Lambda }^{+} \left({\text{u}}\right), {\sigma }_{\rm K}^{-} \left({\text{u}}\right)={\sigma }_{\Lambda }^{-} \left({\text{u}}\right)\mathrm{ and}{ \sigma }_{\rm K}^{+}\left({\text{u}}\right)={\sigma }_{\Lambda }^{+} \left({\text{u}}\right), \forall \mathrm{ u }\in \mathrm{ U}$$

### Definition 2.4

^27^ Assume that $\mathcal{G}$ is the collection of all IVIFSs defined on a finite universal set $U$. A function $S$ from $\mathcal{G} \times \mathcal{G}$ to $\left[0, 1\right]$ is designated as the similarity measure of IVIFSs of $U$, if it meets the requirements listed below:

(S1) $$0 \le S({\rm K}, \Lambda )\le 1$$,

(S2) $$S({\rm K}, \Lambda ) = S(\Lambda , {\rm K})$$,

(S3) $$S({\rm K}, {\rm K}) = 1$$,

(S4) If ${\rm K}\subseteq \Lambda \subseteq {\rm M}$, then $S ({\rm K}, {\rm M})\le \mathrm{min }\{S({\rm K}, \Lambda ),S(\Lambda , {\rm M})\}$ for all ${\rm K},\Lambda ,{\rm M}\in \mathcal{G}$.

### Definition 2.5

^17^ Let $I=\left[\mathrm{0,1}\right]$ and $F:{I}^{4}\to \mathcal{R},$ be defined as:

$$F\left({x}_{1},{x}_{2},{x}_{3},{x}_{4}\right)={cos}^{2}\left[\frac{\pi }{8}\left(\begin{array}{c}{\left|{\text{min}}\left\{{x}_{1},{x}_{4}\right\}-{\text{min}}\left\{{x}_{3},{x}_{2}\right\}\right|}^{2}+\\ {\left|{\text{max}}\left\{{x}_{1},{x}_{4}\right\}-{\text{max}}\left\{{x}_{3},{x}_{2}\right\}\right|}^{2}\end{array}\right)\right]-\frac{1}{4\sqrt{2}}{\left[\begin{array}{c}\left({\left|{x}_{1}-{x}_{3}\right|}^{2}+{\left|{x}_{2}-{x}_{4}\right|}^{2}\right)\\ {\left(2-\left|{x}_{3}+{x}_{4}-{x}_{1}-{x}_{2}\right|\right)}^{2}\end{array}\right]}^\frac{1}{2}.$$

Then $F$ meets the principles listed below.

i. If ${x}_{1},{x}_{2},{x}_{3},{x}_{4}\in \left[\mathrm{0,1}\right]$ and $\left({x}_{1}+{x}_{2}\right),\left({x}_{3}+{x}_{4}\right)\in \left[\mathrm{0,1}\right],$ then $0\le F\left({x}_{1},{x}_{2},{x}_{3},{x}_{4}\right)\le 1.$
ii. If $0\le {a}_{1}\le {a}_{2}\le {a}_{3}\le 1$ and $0\le {b}_{3}\le {b}_{2}\le {b}_{1}\le 1$, then $F\left({a}_{1},{b}_{1},{a}_{3},{b}_{3}\right)\le F\left({a}_{i},{b}_{i},{a}_{i+1},{b}_{i+1}\right)$ for $i=\mathrm{1,2}$.

## Limitations of Current similarity measures of IVIFS

We demonstrate in the subsequent discourse that the similarity measures of IVIFS as delineated in^27,28,31,32^ are ineffectual. Let ${\rm K} = \left\{ {\left\langle {u,{ }\left[ {{ }\gamma_{\rm K}^{ - } \left( u \right),{ }\gamma_{\rm K}^{ + } \left( u \right){ }} \right],\left[ {{ }\sigma_{\rm K}^{ - } \left( u \right),{ }\sigma_{\rm K}^{ + } \left( u \right){ }} \right]} \right\rangle { }\left| {u \in {\text{U}}} \right.} \right\}$ and $\Lambda = \left\{ {\left\langle {u,{ }\left[ {{ }\gamma_{\Lambda }^{ - } \left( u \right),{ }\gamma_{\Lambda }^{ + } \left( u \right){ }} \right],\left[ {{ }\sigma_{\Lambda }^{ - } \left( u \right),{ }\sigma_{\Lambda }^{ + } \left( u \right){ }} \right]} \right\rangle { }\left| {u \in U} \right.} \right\}$ be two IVIFS on $U$.

### Definition 3.1

^27^ Xu’s and Chen similarity measure is defined as:

- $${S}_{1}\left({\rm K}, \Lambda \right)=1-\sqrt[p]{\frac{1}{4n}\left(\sum_{i=1}^{n}\begin{array}{c}{\left|{\gamma }_{\rm K}^{-}\left({u}_{i}\right)- {\gamma }_{\Lambda }^{-}\left({u}_{i}\right)\right|}^{p}+{\left|{\gamma }_{\rm K}^{+}\left({u}_{i}\right)- {\gamma }_{\Lambda }^{+}\left({u}_{i}\right)\right|}^{p}\\ + {\left|{\sigma }_{\rm K}^{-}\left({u}_{i}\right)- {\sigma }_{\Lambda }^{-}\left({u}_{i}\right)\right|}^{p}+{\left|{\sigma }_{\rm K}^{+}\left({u}_{i}\right)- {\sigma }_{\Lambda }^{+}\left({u}_{i}\right)\right|}^{p}\end{array}\right)},$$
- $${S}_{2}\left({\rm K}, \Lambda \right)=1-\sqrt[{\text{p}}]{\frac{1}{{\text{n}}}\left(\sum_{{\text{i}}=1}^{{\text{n}}}\begin{array}{c}max{\left|{\gamma }_{{\text{A}}}^{-}\left({u}_{i}\right)- {\gamma }_{{\text{B}}}^{-}\left({u}_{i}\right)\right|}^{{\text{p}}}+{\left|{\gamma }_{{\text{A}}}^{+}\left({u}_{i}\right)- {\gamma }_{{\text{B}}}^{+}\left({u}_{i}\right)\right|}^{{\text{p}}}\\ + {\left|{\sigma }_{{\text{A}}}^{-}\left({u}_{i}\right)- {\sigma }_{{\text{B}}}^{-}\left({u}_{i}\right)\right|}^{{\text{p}}}+{\left|{\sigma }_{{\text{A}}}^{+}\left({u}_{i}\right)- {\sigma }_{{\text{B}}}^{+}\left({u}_{i}\right)\right|}^{{\text{p}}}\end{array}\right)}$$

The inefficiency of ${S}_{1}$ and ${S}_{2}$ is demonstrated by the following examples 3.2 and 3.3 respectively.

### Example 3.2

 Given the IVIFSs ${\rm K}= \left\{\left[0.30 , 0.40\right], \left[0.50, 0.70\right]\right\}$, $\Lambda = \left\{\left[0.40, 0.50\right], \left[\mathrm{0,50} , 0.70\right]\right\}$ and ${\rm M}= \left\{\left[0.40, 0.50\right] ,\left[0.40, 0.60\right]\right\}$. The application of Definition 3.1 on the above IVIFS yields the following outcomes. ${S}_{1}\left({\rm K}, \Lambda \right)= {S}_{1}\left({\rm K}, {\rm M}\right)=0.9$, where ${\rm K} \ne \Lambda$ and ${\rm K} \ne {\rm M}$. This shows the indistinguishability characteristic of the similarity measure ${S}_{1}$ and hence is ineffective in this case.

### Example 3.3

 Given the IVIFSs ${\rm K}= \left\{\left[0.30, 0.40\right], \left[0.50, 0.70\right]\right\}$, $\Lambda = \left\{\left[0.40, 0.50\right], \left[\mathrm{0,50}, 0.70\right]\right\}$ and ${\rm M}=\left\{\left[0.40, 0.50\right] ,\left[0.40, 0.60\right]\right\}$. The application of Definition 3.1 on the above IVIFS yields the following outcomes:

${S}_{2}\left({\rm K}, \Lambda \right)= {S}_{2}\left({\rm K}, {\rm M}\right)=0.9$, where ${\rm K} \ne \Lambda$ and ${\rm K} \ne {\rm M}$. This shows the indistinguishability characteristic of the similarity measure ${S}_{2}$ and hence is ineffective in this case.

### Definition 3.4

^28^ Wei’s et al*.* similarity measure is defined as:

$${S}_{w}\left({\rm K} , \Lambda \right)= \frac{1}{n}\sum_{i=1}^{n}\frac{2-{\text{min}}\left({\mu }_{i}^{-} , {\nu }_{i}^{-}\right)- {\text{min}}\left({\mu }_{i}^{+} , {\nu }_{i}^{+}\right)}{2-{\text{max}}\left({\mu }_{i}^{-} , {\nu }_{i}^{-}\right)- {\text{max}}\left({\mu }_{i}^{+} , {\nu }_{i}^{+}\right)}$$

where,

$${\mu }_{i}^{-}=\left|{\gamma }_{\rm K}^{-}\left({u}_{i}\right)- {\gamma }_{\Lambda }^{-}\left({u}_{i}\right)\right|, {\mu }_{i}^{+}=\left|{\gamma }_{\rm K}^{+}\left({u}_{i}\right)- {\gamma }_{\Lambda }^{+}\left({u}_{i}\right)\right|$$

and $${\nu }_{i}^{-}=\left|{\sigma }_{\rm K}^{-}\left({u}_{i}\right)- {\sigma }_{\Lambda }^{-}\left({u}_{i}\right)\right|, {\nu }_{i}^{+}=\left|{\sigma }_{\rm K}^{+}\left({u}_{i}\right)- {\sigma }_{\Lambda }^{+}\left({u}_{i}\right)\right|.$$

The invalidity of ${S}_{{\text{W}}}$ is illustrated by the following example 3.5

### Example 3.5

Given the IVIFSs ${\rm K}= \left\{\left[0.30, 0.40\right], \left[0.50, 0.70\right]\right\}$, $\Lambda = \left\{\left[0.40, 0.50\right], \left[\mathrm{0,50}, 0.70\right]\right\}$ and ${\rm M}= \left\{\left[0.40, 0.50\right] ,\left[0.40, 0.60\right]\right\}$. The application of Definition 3.4 on the above IVIFS yields the following outcomes:

${S}_{{\text{W}}}\left({\rm K}, \Lambda \right)= {S}_{{\text{W}}}\left({\rm K}, {\rm M}\right)=0.21$, where ${\rm K} \ne \Lambda$ and ${\rm K} \ne {\rm M}$. This shows the indistinguishability characteristic of the similarity measure ${S}_{{\text{W}}}$ and hence is ineffective in this case.

### Definition 3.6

Dhivya and Sridevi’s similarity measure^31^ is defined as:

$${S}_{D}\left({\rm K} , \Lambda \right)=1- \frac{1}{n}\sum_{i=1}^{n}\left(\begin{array}{c}\frac{1}{2} \left(\begin{array}{c}\left|{\dot{\psi }}_{\rm K}^{-}\left({u}_{i}\right)- {\dot{\psi }}_{\Lambda }^{-}\left({u}_{i}\right)\right|+\\ \left|{\dot{\psi }}_{\rm K}^{+}\left({u}_{i}\right)- {\dot{\psi }}_{\Lambda }^{+}\left({u}_{i}\right)\right|\end{array}\right).\\ \left(1-\frac{{\mu }_{\rm K}\left({u}_{i}\right)+ {\mu }_{\Lambda }\left({u}_{i}\right)}{2}\right)+\\ \left|{\mu }_{\rm K}\left({u}_{i}\right)+ {\mu }_{\Lambda }\left({u}_{i}\right)\right|.\left(\frac{{\mu }_{\rm K}\left({u}_{i}\right)+ {\mu }_{\Lambda }\left({u}_{i}\right)}{2}\right)\end{array}\right),$$

where

$${\psi }_{\rm K}^{-}\left({y}_{i}\right)=\frac{{\gamma }_{\rm K}^{-}\left({u}_{i}\right)+1- {\sigma }_{\rm K}^{-}\left({u}_{i}\right)}{2} , {\psi }_{\Lambda }^{+}\left({y}_{i}\right)=\frac{{\gamma }_{\rm K}^{+}\left({u}_{i}\right)+1- {\sigma }_{\rm K}^{+}\left({u}_{i}\right)}{2}, {\psi }_{\Lambda }^{-}\left({y}_{i}\right)=\frac{{\gamma }_{\Lambda }^{-}\left({u}_{i}\right)+1- {\sigma }_{\Lambda }^{-}\left({u}_{i}\right)}{2} , {\psi }_{\Lambda }^{+}\left({y}_{i}\right)=\frac{{\gamma }_{\Lambda }^{+}\left({u}_{i}\right)+1- {\sigma }_{\Lambda }^{+}\left({u}_{i}\right)}{2} ,$$

$${\mu }_{\rm K}\left({y}_{i}\right)=1- \frac{1}{2} \left({\gamma }_{\rm K}^{-}\left({u}_{i}\right)+ {\gamma }_{\rm K}^{+}\left({u}_{i}\right)+ {\sigma }_{\rm K}^{-}\left({u}_{i}\right)+ {\sigma }_{\rm K}^{+}\left({u}_{i}\right)\right)$$

$${\mu }_{\Lambda }\left({y}_{i}\right)=1- \frac{1}{2} \left({\gamma }_{\Lambda }^{-}\left({u}_{i}\right)+ {\gamma }_{\Lambda }^{+}\left({u}_{i}\right)+ {\sigma }_{\Lambda }^{-}\left({u}_{i}\right)+ {\sigma }_{\Lambda }^{+}\left({u}_{i}\right)\right)$$

The inefficacy of ${S}_{{\text{D}}}$ is illustrated by the following example 3.7.

### Example 3.7

Given the IVIFSs ${\rm K}= \left\{\left[0.30, 0.40\right], \left[0.50, 0.70\right]\right\}$, $\Lambda = \left\{\left[0.40, 0.50\right], \left[\mathrm{0,50}, 0.70\right]\right\}$ and ${\rm M}= \left\{\left[0.40, 0.50\right] ,\left[0.40, 0.60\right]\right\}$. The application of Definition 3.6 on the above IVIFS yields the following outcomes. ${S}_{{\text{D}}}\left({\rm K}, \Lambda \right)= {S}_{{\text{D}}}\left({\rm K}, {\rm M}\right)=1.00$, where ${\rm K} \ne \Lambda$ and ${\rm K} \ne {\rm M}$. This shows the indistinguishability characteristic of the similarity measure ${S}_{{\text{D}}}$ and hence is ineffective in this case.

### Definition 3.8

Luo and Liang’s similarity measure^32^ is given as:

$${S}^{p}\left( {\rm K}, \Lambda \right)=1-{\left\{\frac{1}{2n} \sum_{i=1}^{n}{\left|\frac{\begin{array}{c}{t}_{1}\left[\left({\gamma }_{\rm K}^{-}\left({u}_{i}\right)- {\gamma }_{\Lambda }^{-}\left({u}_{i}\right)\right)+\left({\gamma }_{\rm K}^{+}\left({u}_{i}\right)- {\gamma }_{\Lambda }^{+}\left({u}_{i}\right)\right)\right]\\ -\left[\left({\sigma }_{\rm K}^{-}\left({u}_{i}\right)- {\sigma }_{\Lambda }^{-}\left({u}_{i}\right)\right)+\left({\sigma }_{\rm K}^{+}\left({u}_{i}\right)- {\sigma }_{\Lambda }^{+}\left({u}_{i}\right)\right)\right]\end{array}}{2\left({t}_{1}+1\right)}\right|}^{p}+ {\left|\frac{\begin{array}{c}{t}_{2}\left[\left({\sigma }_{\rm K}^{-}\left({u}_{i}\right)- {\sigma }_{\Lambda }^{-}\left({u}_{i}\right)\right)+\left({\sigma }_{\rm K}^{+}\left({u}_{i}\right)- {\sigma }_{\Lambda }^{+}\left({u}_{i}\right)\right)\right]\\ - \left[\left({\gamma }_{\rm K}^{-}\left({u}_{i}\right)- {\gamma }_{\Lambda }^{-}\left({u}_{i}\right)\right)+\left({\gamma }_{\rm K}^{+}\left({u}_{i}\right)- {\gamma }_{\Lambda }^{+}\left({u}_{i}\right)\right)\right]\end{array}}{2\left({t}_{2}+1\right)}\right|}^{p} \right\}}^\frac{1}{p}.$$

The incapability of ${S}^{p}$ is illustrated by the following example 3.9.

### Example 3.9

Given the IVIFSs ${\rm K}= \left\{\left[0.30, 0.40\right], \left[0.50, 0.70\right]\right\}$, $\Lambda = \left\{\left[0.40, 0.50\right], \left[\mathrm{0,50}, 0.70\right]\right\}$ and ${\rm M}= \left\{\left[0.40, 0.50\right] ,\left[0.40, 0.60\right]\right\}$. The application of Definition 3.8 on the above IVIFS yields the following outcomes. ${S}^{p}\left({\rm K}, \Lambda \right)= {S}^{p}\left({\rm K}, {\rm M}\right)=0.93$, where ${\rm K} \ne \Lambda$ and ${\rm K} \ne {\rm M}$. This shows the indistinguishability characteristic of the similarity measure ${S}^{p}$ and hence is ineffective in this case.

## Proposed similarity measure

In this section, we introduce a novel similarity measure between IVIFSs and prove its structural properties. We also conduct a comparative study to demonstrate the validity of this newly defined similarity measure in comparison to the existing measures discussed in Section III.

### Fundamental characteristics of proposed similarity measure on IVIFS

This section deals with the initiation of the novel similarity measure $\mathcal{T}$ between IVIFS and highlights its fundamental properties.

#### Definition 4.1.1

Let ${\rm K} = \left\{ {\left\langle {u,{ }\left[ {{ }\gamma_{\rm K}^{ - } \left( u \right),{ }\gamma_{\rm K}^{ + } \left( u \right){ }} \right],\left[ {{ }\sigma_{\rm K}^{ - } \left( u \right),{ }\sigma_{\rm K}^{ + } \left( u \right){ }} \right]} \right\rangle { }\left| {u \in U} \right.} \right\},$ and $\Lambda = \left\{ {\left\langle {u,{ }\left[ {{ }\gamma_{\Lambda }^{ - } \left( u \right),{ }\gamma_{\Lambda }^{ + } \left( u \right){ }} \right],\left[ {{ }\sigma_{\Lambda }^{ - } \left( u \right),{ }\sigma_{\Lambda }^{ + } \left( u \right){ }} \right]} \right\rangle { }\left| {u \in U} \right.} \right\}$ be two IVIFS defined on a finite universal set $U= \left\{{u}_{1},{u}_{2},\dots ,{u}_{n}\right\}$. Here, $\left[{\gamma }_{\rm K}^{-}\left(u\right), {\gamma }_{\rm K}^{+}\left(u\right)\right]$ and $\left[{\sigma }_{\rm K}^{-}\left(u\right), {\sigma }_{\rm K}^{+}\left(u\right)\right]$ represent the membership and non-membership degree of IVIFS ${\rm K}$ respectively. In a similar manner, $\left[{\gamma }_{\Lambda }^{-}\left(u\right), {\gamma }_{\Lambda }^{+}\left(u\right)\right]$ and $\left[{\sigma }_{\Lambda }^{-}\left(u\right), {\sigma }_{\Lambda }^{+}\left(u\right)\right]$ are the membership and non-membership degree of IVIFS $\Lambda$ respectively. The similarity measure $\mathcal{T}$ on ${\rm K}$ and $\Lambda$ is defined as follows:

$$\mathcal{T}\left({\rm K},\Lambda \right)=\frac{1}{\lambda }\sum_{i=1}^{n}\left[\begin{array}{c}{cos}^{2}\left\{\frac{\pi }{8}\left(\begin{array}{c}\left|\begin{array}{c}min\left\{{\gamma }_{\rm K}^{-}\left({u}_{i}\right),{\sigma }_{\Lambda }^{-}\left({u}_{i}\right) \right\}+\\ min\left\{{\gamma }_{\rm K}^{+}\left({u}_{i}\right),{\sigma }_{\Lambda }^{+}\left({u}_{i}\right) \right\}- \\ min\left\{{\gamma }_{\Lambda }^{-}\left({u}_{i}\right),{\sigma }_{\rm K}^{-}\left({u}_{i}\right) \right\}\\ +min\left\{{\gamma }_{\Lambda }^{+}\left({u}_{i}\right),{\sigma }_{\rm K}^{+}\left(u\right) \right\}\end{array}\right|+\\ \left|\begin{array}{c}max\left\{{\gamma }_{\rm K}^{-}\left({u}_{i}\right),{\sigma }_{\Lambda }^{-}\left({u}_{i}\right) \right\}\\ +max\left\{{\gamma }_{\rm K}^{+}\left({u}_{i}\right),{\sigma }_{\Lambda }^{+}\left({u}_{i}\right) \right\}- \\ max\left\{{\gamma }_{\Lambda }^{-}\left({u}_{i}\right),{\sigma }_{\rm K}^{-}\left({u}_{i}\right) \right\}\\ +max\left\{{\gamma }_{\Lambda }^{+}\left({u}_{i}\right),{\sigma }_{\rm K}^{+}\left({u}_{i}\right) \right\}\end{array}\right|\end{array}\right)\right\}\\ -\frac{1}{4\sqrt{2}}{\left\{\begin{array}{c}\left(\begin{array}{c}{\left|\begin{array}{c}{\gamma }_{\rm K}^{-}\left({u}_{i}\right)- {\gamma }_{\Lambda }^{-}\left({u}_{i}\right)\\ +{\gamma }_{\rm K}^{+}\left({u}_{i}\right)- {\gamma }_{\Lambda }^{+}\left({u}_{i}\right)\end{array}\right|}^{2}+\\ {\left|\begin{array}{c}{\sigma }_{\rm K}^{-}\left({u}_{i}\right)- {\sigma }_{\Lambda }^{-}\left({u}_{i}\right)\\ +{\sigma }_{\Lambda }^{+}\left({u}_{i}\right)- {\sigma }_{\Lambda }^{+}\left({u}_{i}\right)\end{array}\right|}^{2}\end{array}\right)\\ {\left(2-\left|\begin{array}{c}{\pi }_{\rm K}^{-}\left({u}_{i}\right)- {\pi }_{\Lambda }^{-}\left({u}_{i}\right)+\\ {\pi }_{\rm K}^{+}\left({u}_{i}\right)- {\pi }_{\Lambda }^{+}\left({u}_{i}\right)\end{array}\right|\right)}^{2}\end{array}\right\}}^\frac{1}{2}\end{array}\right]$$

Herein, $$\lambda >0.$$

The validity of the similarity measure $\mathcal{T}$ is illustrated in the following example 4.2.1.

#### Example 4.1.2

Given the IVIFSs ${\rm K}= \left\{\left[0.30, 0.40\right], \left[0.50, \mathrm{0,70}\right]\right\}$, $\Lambda = \left\{\left[0.40, 0.50\right], \left[\mathrm{0,50}, 0.70\right]\right\}$ and ${\rm M}= \left\{\left[0.40, 0.50\right] ,\left[0.40, \mathrm{0,60}\right]\right\}$. The application of Definition 4.1.1 on the above IVIFS yields the following outcomes. $\mathcal{T}\left({\rm K}, \Lambda \right)=0.7,$ and $\mathcal{T}\left({\rm K}, {\rm M}\right)=0.8$, where $\mathrm{A }\ne \mathrm{ B}$ and ${\rm K} \ne {\rm M}$. This shows the distinguishability characteristic of the similarity measure $\mathcal{T}$ and hence is effective in this case.

#### Theorem 4.1.3

Let ${\rm K},\Lambda$ and ${\rm M}$ be any three IVIFSs defined on a universal set $U=\{{u}_{1}, {u}_{2}, {u}_{3},\dots ,{u}_{n}\}$ and $\mathcal{G}$ be the class of all IVIFSs defined on $U$. Then,

$$\mathcal{T}\left({\rm K},\Lambda \right)=\frac{1}{\lambda }\sum_{i=1}^{n}\left[\begin{array}{c}{cos}^{2}\left\{\frac{\pi }{8}\left(\begin{array}{c}\left|\begin{array}{c}min\left\{{\gamma }_{\rm K}^{-}\left({u}_{i}\right),{\sigma }_{\Lambda }^{-}\left({u}_{i}\right) \right\}+\\ min\left\{{\gamma }_{\rm K}^{+}\left({u}_{i}\right),{\sigma }_{\Lambda }^{+}\left({u}_{i}\right) \right\}- \\ min\left\{{\gamma }_{\Lambda }^{-}\left({u}_{i}\right),{\sigma }_{\rm K}^{-}\left({u}_{i}\right) \right\}\\ +min\left\{{\gamma }_{\Lambda }^{+}\left({u}_{i}\right),{\sigma }_{\rm K}^{+}\left(u\right) \right\}\end{array}\right|+\\ \left|\begin{array}{c}max\left\{{\gamma }_{\rm K}^{-}\left({u}_{i}\right),{\sigma }_{\Lambda }^{-}\left({u}_{i}\right) \right\}\\ +max\left\{{\gamma }_{\rm K}^{+}\left({u}_{i}\right),{\sigma }_{\Lambda }^{+}\left({u}_{i}\right) \right\}- \\ max\left\{{\gamma }_{\Lambda }^{-}\left({u}_{i}\right),{\sigma }_{\rm K}^{-}\left({u}_{i}\right) \right\}\\ +max\left\{{\gamma }_{\Lambda }^{+}\left({u}_{i}\right),{\sigma }_{\rm K}^{+}\left({u}_{i}\right) \right\}\end{array}\right|\end{array}\right)\right\}-\frac{1}{4\sqrt{2}}{\left\{\begin{array}{c}\left(\begin{array}{c}{\left|\begin{array}{c}{\gamma }_{\rm K}^{-}\left({u}_{i}\right)- {\gamma }_{\Lambda }^{-}\left({u}_{i}\right)\\ +{\gamma }_{\rm K}^{+}\left({u}_{i}\right)- {\gamma }_{\Lambda }^{+}\left({u}_{i}\right)\end{array}\right|}^{2}+\\ {\left|\begin{array}{c}{\sigma }_{\rm K}^{-}\left({u}_{i}\right)- {\sigma }_{\Lambda }^{-}\left({u}_{i}\right)\\ +{\sigma }_{\Lambda }^{+}\left({u}_{i}\right)- {\sigma }_{\Lambda }^{+}\left({u}_{i}\right)\end{array}\right|}^{2}\end{array}\right)\\ {\left(2-\left|\begin{array}{c}{\pi }_{\rm K}^{-}\left({u}_{i}\right)- {\pi }_{\Lambda }^{-}\left({u}_{i}\right)+\\ {\pi }_{\rm K}^{+}\left({u}_{i}\right)- {\pi }_{\Lambda }^{+}\left({u}_{i}\right)\end{array}\right|\right)}^{2}\end{array}\right\}}^\frac{1}{2}\end{array}\right]$$

admits all the structural properties of a similarity measure.

#### Proof

Let ${\rm K} = \left\{ {\left\langle {u,{ }\left[ {{ }\gamma_{\rm K}^{ - } \left( u \right),{ }\gamma_{\rm K}^{ + } \left( u \right){ }} \right],\left[ {{ }\sigma_{\rm K}^{ - } \left( u \right),{ }\sigma_{\rm K}^{ + } \left( u \right){ }} \right]} \right\rangle { }\left| {u \in {\text{U}}} \right.} \right\},{ }\Lambda = \left\{ {\left\langle {u,{ }\left[ {{ }\gamma_{\Lambda }^{ - } \left( u \right),{ }\gamma_{\Lambda }^{ + } \left( u \right){ }} \right],\left[ {{ }\sigma_{\Lambda }^{ - } \left( u \right),{ }\sigma_{\Lambda }^{ + } \left( u \right){ }} \right]} \right\rangle { }\left| {u \in U} \right.} \right\}$ and ${\rm M} = \left\{ {\left\langle {u,{ }\left[ {{ }\gamma_{\rm M}^{ - } \left( u \right),{ }\gamma_{\rm M}^{ + } \left( u \right){ }} \right],\left[ {{ }\sigma_{\rm M}^{ - } \left( u \right),{ }\sigma_{\rm M}^{ + } \left( u \right){ }} \right]} \right\rangle \left| {u \in U} \right.} \right\}$ be the interval valued intuitionistic fuzzy sets on $U$.

**(S1)** *Case I*

First, we solve this property for ${\mathcal{T}}^{-}$ which represents the lower membership and non-membership degrees of IVIFS ${\rm K}$ and $\Lambda$.

Since ${\gamma }_{\rm K}^{-}\left(u\right), {\gamma }_{\Lambda }^{-}\left(u\right), {\sigma }_{\rm K}^{-}\left(u\right), {\sigma }_{\Lambda }^{-}\left(u\right), \in \left[0, 1\right]$ and ${\gamma }_{\rm K}^{-}\left(u\right)+ {\sigma }_{\rm K}^{-}\left(u\right) , {\gamma }_{\Lambda }^{-}\left(u\right)+ {\sigma }_{\Lambda }^{-}\left(u\right) \in \left[\mathrm{0,1}\right]$. The subsequent inequality results from considering Definition 2.5 (1):

$$0\le F\left({\gamma }_{\rm K}^{-}\left({u}_{i}\right), {\gamma }_{\Lambda }^{-}\left({u}_{i}\right), {\sigma }_{\rm K}^{-}\left({u}_{i}\right), {\sigma }_{\Lambda }^{-}\left({u}_{i}\right)\right) \le 1, \forall i=1, 2, 3, \dots , n.$$

This implies that

$$0\le \left[\begin{array}{c}{cos}^{2}\left\{\frac{\pi }{8}\left(\begin{array}{c}\left|\begin{array}{c}min\left\{{\gamma }_{\rm K}^{-}\left({u}_{i}\right),{\sigma }_{\Lambda }^{-}\left({u}_{i}\right) \right\}\\ -min\left\{{\gamma }_{\Lambda }^{-}\left({u}_{i}\right),{\sigma }_{\rm K}^{-}\left({u}_{i}\right) \right\}\end{array}\right|+\\ \left|\begin{array}{c}max\left\{{\gamma }_{\rm K}^{-}\left({u}_{i}\right),{\sigma }_{\Lambda }^{-}\left({u}_{i}\right) \right\}\\ -max\left\{{\gamma }_{\Lambda }^{-}\left({u}_{i}\right),{\sigma }_{\rm K}^{-}\left({u}_{i}\right) \right\} \end{array}\right|\end{array}\right)\right\}-\frac{1}{4\sqrt{2}}{\left\{\begin{array}{c}\left(\begin{array}{c}{\left|{\dot{\gamma }}_{\rm K}^{-}\left({u}_{i}\right)- {\dot{\gamma }}_{\Lambda }^{-}\left({u}_{i}\right)\right|}^{2}\\ +{\left|{\sigma }_{\rm K}^{-}\left(u\right)- {\sigma }_{\Lambda }^{-}\left({u}_{i}\right)\right|}^{2}\end{array}\right)\\ {\left(2-\left|{\pi }_{\rm K}^{-}\left({u}_{i}\right)- {\pi }_{\Lambda }^{-}\left({u}_{i}\right)\right|\right)}^{2}\end{array}\right\}}^\frac{1}{2}\end{array}\right] \le 1,$$

which further leads to

$$0\le \frac{1}{\lambda } \sum_{i=1}^{n}\left[\begin{array}{c}{cos}^{2}\left\{\frac{\pi }{8}\left(\begin{array}{c}\left|\begin{array}{c}min\left\{{\gamma }_{\rm K}^{-}\left({u}_{i}\right),{\sigma }_{\Lambda }^{-}\left({u}_{i}\right) \right\}\\ -min\left\{{\gamma }_{\Lambda }^{-}\left({u}_{i}\right),{\sigma }_{\rm K}^{-}\left({u}_{i}\right) \right\}\end{array}\right|+\\ \left|\begin{array}{c}max\left\{{\gamma }_{\rm K}^{-}\left({u}_{i}\right),{\sigma }_{\Lambda }^{-}\left({u}_{i}\right) \right\}\\ -max\left\{{\gamma }_{\Lambda }^{-}\left({u}_{i}\right),{\sigma }_{\rm K}^{-}\left({u}_{i}\right) \right\} \end{array}\right|\end{array}\right)\right\}-\frac{1}{4\sqrt{2}}{\left\{\begin{array}{c}\left(\begin{array}{c}{\left|{\dot{\gamma }}_{\rm K}^{-}\left({u}_{i}\right)- {\dot{\gamma }}_{\Lambda }^{-}\left({u}_{i}\right)\right|}^{2}\\ +{\left|{\sigma }_{\rm K}^{-}\left(u\right)- {\sigma }_{\Lambda }^{-}\left({u}_{i}\right)\right|}^{2}\end{array}\right)\\ {\left(2-\left|{\pi }_{\rm K}^{-}\left({u}_{i}\right)- {\pi }_{\Lambda }^{-}\left({u}_{i}\right)\right|\right)}^{2}\end{array}\right\}}^\frac{1}{2}\end{array}\right] \le 1.$$

$$\mathrm{Hence }0\le {\mathcal{T}}^{-}({\rm K}, \Lambda )\le$$ 1 (1).

Case 2: One can establish the above inequality for ${\mathcal{T}}^{+}$ which represents the upper membership and non-membership degrees of IVIFS ${\rm K}$ and $\Lambda$.

As a result of this, we obtain:

2 $$0\le {\mathcal{T}}^{+}({\rm K}, \Lambda )\le 1$$

By comparing (1) and (2), we obtain the following inequality

$$0\le \mathcal{T}({\rm K}, \Lambda )\le$$ 1.

**(S2) Case 1:** First, we solve this property for the lower case ${\mathcal{T}}^{-}$

Consider the following

$${\zeta }_{i}=\begin{array}{c}\left|min\left\{{\gamma }_{\rm K}^{-}\left({u}_{i}\right),{\sigma }_{\Lambda }^{-}\left({u}_{i}\right) \right\}-min\left\{{\gamma }_{\Lambda }^{-}\left({u}_{i}\right),{\sigma }_{\rm K}^{-}\left({u}_{i}\right) \right\}\right|\\+ \left|max\left\{{\gamma }_{\rm K}^{-}\left({u}_{i}\right),{\sigma }_{\Lambda }^{-}\left({u}_{i}\right) \right\}-max\left\{{\gamma }_{\Lambda }^{-}\left({u}_{i}\right),{\sigma }_{\rm K}^{-}\left({u}_{i}\right) \right\}\right|\end{array}$$

and

$${\rho }_{i}=\left({\left|{\gamma }_{\rm K}^{-}\left({u}_{i}\right)- {\gamma }_{\Lambda }^{-}\left({u}_{i}\right)\right|}^{2}+{\left|{\sigma }_{\rm K}^{-}\left({u}_{i}\right)- {\sigma }_{\Lambda }^{-}\left({u}_{i}\right)\right|}^{2}\right){\left(2-\left|{\pi }_{\rm K}^{-}\left({u}_{i}\right)- {\pi }_{\Lambda }^{-}\left({u}_{i}\right)\right|\right)}^{2}$$

Then we have

$${\mathcal{T}}^{-}\left({\rm K},\Lambda \right)=\frac{1}{\lambda }\sum_{i=1}^{n}\left[{cos}^{2}\left(\frac{\pi }{8}{\zeta }_{i}\right)-\frac{1}{4\sqrt{2}}\sqrt{{\rho }_{i}}\right]$$

In view inequality (1), ${\mathcal{T}}^{-}\left({\rm K},\Lambda \right)$ can be express as follows

$$0\le {cos}^{2}\left(\frac{\pi }{8}{\zeta }_{i}\right)-\frac{1}{4\sqrt{2}}\sqrt{{\rho }_{i}}\le 1$$

If $${\mathcal{T}}^{-}\left({\rm K},\Lambda \right)=1$$, then

$${cos}^{2}\left(\frac{\pi }{8}{\zeta }_{i}\right)-\frac{1}{4\sqrt{2}}\sqrt{{\rho }_{i}}=1$$

$${cos}^{2}\left(\frac{\pi }{8}{\zeta }_{i}\right)=1+\frac{1}{4\sqrt{2}}\sqrt{{\rho }_{i}}$$

Since $\frac{1}{2}\le {cos}^{2}\left(\frac{\pi }{8}{\zeta }_{i}\right)\le 1$, this implies that ${\rho }_{i}=0$, it follows that

$$\left({\left|{\gamma }_{\rm K}^{-}\left({u}_{i}\right)- {\gamma }_{\Lambda }^{-}\left({u}_{i}\right)\right|}^{2}+{\left|{\sigma }_{\rm K}^{-}\left({u}_{i}\right)- {\sigma }_{\Lambda }^{-}\left({u}_{i}\right)\right|}^{2}\right){\left(2-\left|{\pi }_{\rm K}^{-}\left({u}_{i}\right)- {\pi }_{\Lambda }^{-}\left({u}_{i}\right)\right|\right)}^{2}=0$$

This shows that

$${\gamma }_{\rm K}^{-}\left({u}_{i}\right)= {\gamma }_{\Lambda }^{-}\left({u}_{i}\right),{\sigma }_{\rm K}^{-}\left({u}_{i}\right)= {\sigma }_{\Lambda }^{-}\left({u}_{i}\right).$$

Consequently, we obtain that if ${\mathcal{T}}^{-}\left({\rm K},\Lambda \right)=1$, then ${\rm K}=\Lambda$.

Conversely, if ${\rm K}=\Lambda$, then clearly ${\mathcal{T}}^{-}\left({\rm K},\Lambda \right)=1$.

*Case 2:* One can establish the above equality for upper case ${\mathcal{T}}^{+}$

Consequently,

$$\mathcal{T}\left({\rm K}, {\rm K}\right)=1$$

Furthermore, the condition (S3) is readily demonstrable.

**(S4)** *Case 1:* First, we solve this property for the lower case ${\mathcal{T}}^{-}$

Let ${\rm K}\subseteq \Lambda \subseteq {\rm M}$ then, $0\le {\gamma }_{\rm K}^{-}\left({\text{u}}\right) \le {\gamma }_{\Lambda }^{-}\left({\text{u}}\right) \le {\gamma }_{\rm M}^{-}\left({\text{u}}\right) \le 1$ and $0\le {\sigma }_{\rm K}^{-}\left(u\right) \le {\sigma }_{\Lambda }^{-}\left(u\right) \le {\sigma }_{\rm M}^{-}\left(u\right) \le 1$. By applying Definition 2.5 (2), the subsequent inequalities result:

$$F\left({\gamma }_{\rm K}^{-}\left({u}_{i}\right), {\sigma }_{\rm K}^{-}\left({u}_{i}\right){, \gamma }_{\rm M}^{-}\left({u}_{i}\right),{\sigma }_{\rm M}^{-}\left({u}_{i}\right)\right)\ge F\left({\gamma }_{\rm K}^{-}\left({u}_{i}\right), {\sigma }_{\rm K}^{-}\left({u}_{i}\right){, \gamma }_{\Lambda }^{-}\left({u}_{i}\right),{\sigma }_{\Lambda }^{-}\left({u}_{i}\right)\right),$$

3 $$\mathrm{where\, i \,ranges \,from \,1,2},\dots n$$

$$F\left({\gamma }_{\rm K}^{-}\left({u}_{i}\right), {\sigma }_{\rm K}^{-}\left({u}_{i}\right){, \gamma }_{\rm M}^{-}\left({u}_{i}\right),{\sigma }_{\rm M}^{-}\left({u}_{i}\right)\right)\ge F\left({\gamma }_{\Lambda }^{-}\left({u}_{i}\right), {\sigma }_{\Lambda }^{-}\left({u}_{i}\right){, \gamma }_{\rm M}^{-}\left({u}_{i}\right),{\sigma }_{\rm M}^{-}\left({u}_{i}\right)\right),$$

4 $$\mathrm{where\, i \,ranges \,from \,1,2},\dots n$$

Then application of 4.1.1 in (3) gives that

$$\left[\begin{array}{c}{cos}^{2}\left\{\frac{\pi }{8}\left(\begin{array}{c}\left|\begin{array}{c}min\left\{{\gamma }_{\rm K}^{-}\left({u}_{i}\right),{\sigma }_{\rm M}^{-}\left({u}_{i}\right) \right\}\\ -min\left\{{\gamma }_{\rm M}^{-}\left({u}_{i}\right),{\sigma }_{\rm K}^{-}\left({u}_{i}\right) \right\}\end{array}\right|+\\ \left|\begin{array}{c}max\left\{{\gamma }_{\rm K}^{-}\left({u}_{i}\right),{\sigma }_{\rm M}^{-}\left({u}_{i}\right) \right\}\\ -max\left\{{\gamma }_{\rm M}^{-}\left({u}_{i}\right),{\sigma }_{\rm K}^{-}\left({u}_{i}\right) \right\} \end{array}\right|\end{array}\right)\right\}\\ -\frac{1}{4\sqrt{2}}{\left\{\begin{array}{c}\left(\begin{array}{c}{\left|{\gamma }_{\rm K}^{-}\left({u}_{i}\right)- {\gamma }_{\rm M}^{-}\left({u}_{i}\right)\right|}^{2}\\ +{\left|{\sigma }_{\rm K}^{-}\left(u\right)- {\sigma }_{\rm M}^{-}\left({u}_{i}\right)\right|}^{2}\end{array}\right)\\ {\left(2-\left|{\pi }_{\rm K}^{-}\left({u}_{i}\right)- {\pi }_{\rm M}^{-}\left({u}_{i}\right)\right|\right)}^{2}\end{array}\right\}}^\frac{1}{2}\end{array}\right]\ge \left[\begin{array}{c}{cos}^{2}\left\{\frac{\pi }{8}\left(\begin{array}{c}\left|\begin{array}{c}min\left\{{\gamma }_{\rm K}^{-}\left({u}_{i}\right),{\sigma }_{\Lambda }^{-}\left({u}_{i}\right) \right\}\\ -min\left\{{\gamma }_{\Lambda }^{-}\left({u}_{i}\right),{\sigma }_{\rm K}^{-}\left({u}_{i}\right) \right\}\end{array}\right|+\\ \left|\begin{array}{c}max\left\{{\gamma }_{\rm K}^{-}\left({u}_{i}\right),{\sigma }_{\Lambda }^{-}\left({u}_{i}\right) \right\}\\ -max\left\{{\gamma }_{\Lambda }^{-}\left({u}_{i}\right),{\sigma }_{\rm K}^{-}\left({u}_{i}\right) \right\} \end{array}\right|\end{array}\right)\right\}\\ -\frac{1}{4\sqrt{2}}{\left\{\begin{array}{c}\left(\begin{array}{c}{\left|{\dot{\gamma }}_{\rm K}^{-}\left({u}_{i}\right)- {\dot{\gamma }}_{\Lambda }^{-}\left({u}_{i}\right)\right|}^{2}\\ +{\left|{\sigma }_{\rm K}^{-}\left(u\right)- {\sigma }_{\Lambda }^{-}\left({u}_{i}\right)\right|}^{2}\end{array}\right)\\ {\left(2-\left|{\pi }_{\rm K}^{-}\left({u}_{i}\right)- {\pi }_{\Lambda }^{-}\left({u}_{i}\right)\right|\right)}^{2}\end{array}\right\}}^\frac{1}{2}\end{array}\right]$$

Further, we obtain the following:

$$\frac{1}{\lambda } \sum_{i=1}^{n}\left[\begin{array}{c}{cos}^{2}\left\{\frac{\pi }{8}\left(\begin{array}{c}\left|\begin{array}{c}min\left\{{\gamma }_{\rm K}^{-}\left({u}_{i}\right),{\sigma }_{\rm M}^{-}\left({u}_{i}\right) \right\}\\ -min\left\{{\gamma }_{\rm M}^{-}\left({u}_{i}\right),{\sigma }_{\rm K}^{-}\left({u}_{i}\right) \right\}\end{array}\right|+\\ \left|\begin{array}{c}max\left\{{\gamma }_{\rm K}^{-}\left({u}_{i}\right),{\sigma }_{\rm M}^{-}\left({u}_{i}\right) \right\}\\ -max\left\{{\gamma }_{\rm M}^{-}\left({u}_{i}\right),{\sigma }_{\rm K}^{-}\left({u}_{i}\right) \right\} \end{array}\right|\end{array}\right)\right\}\\ -\frac{1}{4\sqrt{2}}{\left\{\begin{array}{c}\left(\begin{array}{c}{\left|{\gamma }_{\rm K}^{-}\left({u}_{i}\right)- {\gamma }_{\rm M}^{-}\left({u}_{i}\right)\right|}^{2}\\ +{\left|{\sigma }_{\rm K}^{-}\left(u\right)- {\sigma }_{\rm M}^{-}\left({u}_{i}\right)\right|}^{2}\end{array}\right)\\ {\left(2-\left|{\pi }_{\rm K}^{-}\left({u}_{i}\right)- {\pi }_{\rm M}^{-}\left({u}_{i}\right)\right|\right)}^{2}\end{array}\right\}}^\frac{1}{2}\end{array}\right]\le \frac{1}{\lambda } \sum_{i=1}^{n}\left[\begin{array}{c}{cos}^{2}\left\{\frac{\pi }{8}\left(\begin{array}{c}\left|\begin{array}{c}min\left\{{\gamma }_{\rm K}^{-}\left({u}_{i}\right),{\sigma }_{\Lambda }^{-}\left({u}_{i}\right) \right\}\\ -min\left\{{\gamma }_{\Lambda }^{-}\left({u}_{i}\right),{\sigma }_{\rm K}^{-}\left({u}_{i}\right) \right\}\end{array}\right|+\\ \left|\begin{array}{c}max\left\{{\gamma }_{\rm K}^{-}\left({u}_{i}\right),{\sigma }_{\Lambda }^{-}\left({u}_{i}\right) \right\}\\ -max\left\{{\gamma }_{\Lambda }^{-}\left({u}_{i}\right),{\sigma }_{\rm K}^{-}\left({u}_{i}\right) \right\} \end{array}\right|\end{array}\right)\right\}\\ -\frac{1}{4\sqrt{2}}{\left\{\begin{array}{c}\left(\begin{array}{c}{\left|{\dot{\gamma }}_{\rm K}^{-}\left({u}_{i}\right)- {\dot{\gamma }}_{\Lambda }^{-}\left({u}_{i}\right)\right|}^{2}\\ +{\left|{\sigma }_{\rm K}^{-}\left(u\right)- {\sigma }_{\Lambda }^{-}\left({u}_{i}\right)\right|}^{2}\end{array}\right)\\ {\left(2-\left|{\pi }_{\rm K}^{-}\left({u}_{i}\right)- {\pi }_{\Lambda }^{-}\left({u}_{i}\right)\right|\right)}^{2}\end{array}\right\}}^\frac{1}{2}\end{array}\right]$$

This implies that

5 $${\mathcal{T}}^{-}\left({\rm K}, {\rm M}\right)\le {\mathcal{T}}^{-}\left( {\rm K},\Lambda \right)$$

Then application of 4.1.1 in (3) and by adopting the above procedure we get

6 $${\mathcal{T}}^{-}\left({\rm K}, {\rm M}\right)\le {\mathcal{T}}^{-}\left( \Lambda , {\rm M}\right)$$

By comparing the inequality (5) and (6), we get

7 $${\mathcal{T}}^{-}\left({\rm K}, {\rm M}\right)\le min\left\{{\mathcal{T}}^{-}\left( {\rm K},\Lambda \right),{\mathcal{T}}^{-}\left( \Lambda , {\rm M}\right)\right\}$$

*Case 2:* One can establish above inequality for upper case ${\mathcal{T}}^{+}$.

8 $${\mathcal{T}}^{+}\left({\rm K}, {\rm M}\right)\le min\left\{{\mathcal{T}}^{+}\left( {\rm K},\Lambda \right),{\mathcal{T}}^{+}\left( \Lambda , {\rm M}\right)\right\}$$

The comparison of (7) and (8) gives that

$$\mathcal{T}\left({\rm K}, {\rm M}\right)\le {\text{min}}\{\mathcal{T}\left({\rm K}, \Lambda \right), \mathcal{T}(\Lambda , {\rm M})$$

*Remark 4.1.4* Notably, the similarity measure specified in^17^ is transformed into a specific case of the Definition 4.1.1 by taking ${\gamma }_{\rm K}^{-}= {\gamma }_{\rm K}^{+} , { \gamma }_{\Lambda }^{-}= {\gamma }_{\Lambda }^{+}, {\sigma }_{\rm K}^{-}= {\sigma }_{\rm K}^{+} , { \sigma }_{\Lambda }^{-}= {\sigma }_{\Lambda }^{+}$.

### Comparative analysis

In the subsequent discourse, we establish a comparison with pre-existing similarity measures in the IVIFS setting in order to illustrate the reliability and practicability of the suggested similarity measure. Let us consider IVIFSs $= \left\{\left[0.3 , 0.4\right], \left[0.5 , \mathrm{0,7}\right]\right\}$, $\Lambda = \left\{\left[0.4, 0.5\right], \left[\mathrm{0,5} , 0.7\right]\right\}$ and ${\rm M}= \left\{\left[0.4 , 0.5\right] ,\left[0.4 , \mathrm{0,6}\right]\right\}$.

In Table 1, upon comparing the both columns of the known similarity measures ${S}_{1 },{S}_{2},{ S}_{w}$ and ${S}^{P}$, it becomes evident that these similarity measures are not reasonable as they do not satisfy the following relation.$S\left({\rm K}, \Lambda \right)=S({\rm K},{\rm M})$, while and $\Lambda \ne {\rm M}$ indicating that the existing similarity measures are not reasonable. Moreover, ${S}_{D}\left({\rm K}, \Lambda \right)={S}_{D}\left({\rm K}, {\rm M}\right)=1.00$ while $\Lambda \ne {\rm M}$, showing that t ${S}_{D}$ fails to meet the condition (S3) of Definition 2.4. However, the proposed similarity measure $\mathcal{T}$ effectively addresses all these cases.

**Table 1:** Comparison of similarity measures within the framework of IVIFS.

	$(A, B)$	$(A, C)$
${S}_{1}$	$0.9$	$0.9$
${S}_{2}$	$0.9$	$0.9$
${S}_{W}$	$0.21$	$0.21$
${S}_{D}$	$1.00$	$1.00$
${S}^{P}$	$0.93$	$0.93$
$\mathcal{T}$	$0.72$	$0.80$

## Utilization of the proposed IVIF similarity measure in MCDM context

This section provides a method to address MCDM issues in order to demonstrate the significance of the suggested similarity measure T within the IVIF context.

Denote the collection of different alternatives as $\left\{{\rm K}_{1}, {\rm K}_{2},{\rm K}_{3},\dots ,{\rm K}_{m}\right\}$ and denote the collection of attributes as $X=\left\{{\epsilon }_{1},{\epsilon }_{2},\dots ,{\epsilon }_{n}\right\}$. Suppose $M= {\left[\left(\left[{\gamma }_{ij}^{-},{\gamma }_{ij}^{+}\right], \left[{\sigma }_{ij}^{-},{\sigma }_{ij}^{+}\right]\right)\right]}_{m\times n}$ is an $m\times n$ IVIF decision matrix, where $0 \le {\upgamma }_{ij}^{-}(\epsilon )+ {\upsigma }_{ij}^{-}(\epsilon ), {\upgamma }_{ij}^{+}(\epsilon )+ {\upsigma }_{ij}^{+}(\epsilon )\le 1$.

Let

$$\Lambda =\left\{\begin{array}{c}\langle {\epsilon }_{1},\left[ {\gamma }_{\Lambda }^{-}\left({\epsilon }_{1}\right), {\gamma }_{\Lambda }^{+}\left({\epsilon }_{1}\right) \right],\left[ {\sigma }_{\Lambda }^{-}\left({\epsilon }_{1}\right), {\sigma }_{\Lambda }^{+}\left({\epsilon }_{1}\right) \right] \rangle , \dots , \\ \langle {\epsilon }_{n},\left[ {\gamma }_{\Lambda }^{-}\left({\epsilon }_{n}\right), {\gamma }_{\Lambda }^{+}\left({\epsilon }_{n}\right) \right],\left[ {\sigma }_{\Lambda }^{-}\left({\epsilon }_{n}\right), {\sigma }_{\Lambda }^{+}\left({\epsilon }_{n}\right) \right] \rangle :{\epsilon }_{j}\in X\end{array}\right\}$$

represent a test sample as categorized by the IVIFS. The method that is utilized to resolve the MCDM challenge within the IVIF paradigm is constructed as follows:

*Step 1.*

Convert the decision matrix $M$ into a normalized matrix $S= {\left({s}_{ij}\right)}_{m\times n}$ (if necessary), where ${S}_{ij}$ is computed by the following equation:

$${S}_{ij}= \left\{\begin{array}{c}\left(\left[{\gamma }_{ij}^{-} ,{\gamma }_{ij}^{+}\right] , \left[{\sigma }_{ij}^{-} ,{\sigma }_{ij}^{+}\right] \right);for\, benefit \,type \,criteria\\ \left( \left[{\sigma }_{ij}^{-} ,{\sigma }_{ij}^{+}\right] ,\left[{\gamma }_{ij}^{-} ,{\gamma }_{ij}^{+}\right] \right);for \,loss \,type \,criteria\end{array}\right.$$

*Step 2.*

Calculate the similarity measures $\mathcal{T}\left({\rm K}_{i}, \Lambda \right)$ between ${\rm K}_{i},$ where $i=\mathrm{1,2},3,\dots ,m,$ and $\Lambda$ as follows:

$$\mathcal{T}\left({\rm K}_{i},\Lambda \right)=\frac{1}{\lambda }\sum_{i=1}^{n}\left[\begin{array}{c}{cos}^{2}\left\{\frac{\pi }{8}\left(\begin{array}{c}\left|\begin{array}{c}min\left\{{\gamma }_{\rm K}^{-}\left({\epsilon }_{ij}\right),{\sigma }_{\Lambda }^{-}\left({\epsilon }_{j}\right) \right\}+\\ min\left\{{\gamma }_{\rm K}^{+}\left({\epsilon }_{ij}\right),{\sigma }_{\Lambda }^{+}\left({\epsilon }_{j}\right) \right\}- \\ min\left\{{\gamma }_{\Lambda }^{-}\left({\epsilon }_{j}\right),{\sigma }_{\rm K}^{-}\left({\epsilon }_{ij}\right) \right\}\\ +min\left\{{\gamma }_{\Lambda }^{+}\left({\epsilon }_{j}\right),{\sigma }_{\rm K}^{+}\left({\epsilon }_{ij}\right) \right\}\end{array}\right|+\\ \left|\begin{array}{c}max\left\{{\gamma }_{\rm K}^{-}\left({\epsilon }_{ij}\right),{\sigma }_{\Lambda }^{-}\left({\epsilon }_{j}\right) \right\}\\ +max\left\{{\gamma }_{\rm K}^{+}\left({\epsilon }_{ij}\right),{\sigma }_{\Lambda }^{+}\left({\epsilon }_{j}\right) \right\}- \\ max\left\{{\gamma }_{\Lambda }^{-}\left({\epsilon }_{j}\right),{\sigma }_{\rm K}^{-}\left({\epsilon }_{ij}\right) \right\}\\ +max\left\{{\gamma }_{\Lambda }^{+}\left({\epsilon }_{j}\right),{\sigma }_{\rm K}^{+}\left({\epsilon }_{ij}\right) \right\}\end{array}\right|\end{array}\right)\right\}-\frac{1}{4\sqrt{2}}{\left\{\begin{array}{c}\left(\begin{array}{c}{\left|\begin{array}{c}{\gamma }_{\rm K}^{-}\left({\epsilon }_{ij}\right)- {\gamma }_{\Lambda }^{-}\left({\epsilon }_{j}\right)\\ +{\gamma }_{\rm K}^{+}\left({\epsilon }_{ij}\right)- {\gamma }_{\Lambda }^{+}\left({\epsilon }_{j}\right)\end{array}\right|}^{2}+\\ {\left|\begin{array}{c}{\sigma }_{\rm K}^{-}\left({\epsilon }_{ij}\right)- {\sigma }_{\Lambda }^{-}\left({\epsilon }_{j}\right)\\ +{\sigma }_{\Lambda }^{+}\left({\epsilon }_{ij}\right)- {\sigma }_{\Lambda }^{+}\left({\epsilon }_{j}\right)\end{array}\right|}^{2}\end{array}\right)\\ {\left(2-\left|\begin{array}{c}{\pi }_{\rm K}^{-}\left({\epsilon }_{ij}\right)- {\pi }_{\Lambda }^{-}\left({\epsilon }_{j}\right)+\\ {\pi }_{\rm K}^{+}\left({\epsilon }_{ij}\right)- {\pi }_{\Lambda }^{+}\left({\epsilon }_{j}\right)\end{array}\right|\right)}^{2}\end{array}\right\}}^\frac{1}{2}\end{array}\right]$$

*Step 3.*

Rank each alternative and select the one with the highest value.

### Selecting the best renewable energy source to reduce energy crises

Energy is necessary for our continued survival and plays a role in almost every aspect of our lives. If we did not have access to energy, we would be considerably limited in our ability to enjoy the conveniences and luxuries of modern life. This is due to the fact that the definition of energy insecurity changes depending on whether a country meets its energy requirements through self-production of energy, imports of energy from other countries, or exports energy to other countries to fulfill its needs. Energy security can be conceptualized as the state of perpetually possessing readily available, economically viable, and accessible energy. This phenomenon is observed in nations that have achieved full economic autonomy. The limited availability of readily available electricity has caused a range of complications that extend throughout Asia, including Pakistan.

Pakistan's energy sector is currently facing a crisis due to a significant shortfall in meeting the increasing energy demands that have accumulated over the past few decades. The demand for energy is increasing rapidly as a result of population growth, urbanization, and industrialization, but the supply of traditional energy sources is insufficient. The energy shortage has resulted in frequent power blackouts, which have hindered economic development, disrupted everyday routines, and obstructed technical advancements. The energy crisis, which is the main cause of economic depletion, has a significant impact on Pakistan's economy. The current crisis originated from a shift in fuel composition that occurred twenty years ago, during which electricity generation increasingly depended on imported furnace oil rather than hydropower. The rise in power generation costs, along with the significant line losses, has necessitated tariff expeditions, resulting in financial losses for power generation, transmission, and distribution corporations. On the other hand, the widespread concern that the world's fossil fuel sources will run out in the near future and that the price of energy will continue to slowly rise is a key issue in today's world. Developing countries may finally be able to find a solution to their long-standing energy problems with the help of renewable energy sources and technology. Given the enormity of Pakistan’s present energy challenge, switching to renewable energy sources may prove to be the most time- and cost-efficient way to solve the problem.

Renewable energy sources are inexhaustible and beneficial to the environment. Biofuels (e.g., ethanol, biodiesel), geothermal energy (by harnessing the thermal energy of water or vapor to drive turbines that generate electricity), organic matter (e.g., dung, wood, vegetation), wind, and oceanic waves are all examples of renewable energy sources. The implementation of renewable energy sources is critical in the prevention and management of energy crises. The significance of renewable energy lies in its capacity to meet the growing demand for electricity while preventing the depletion of finite natural resources. Reduced reliance on foreign fuels also reduces the potential for environmental problems like gasoline spills and emissions. Our long-term energy needss could be met by renewable sources, provided we have enough of them and use a variety of fuels. In this article, we propose a step-by-step procedure for choosing the best renewable energy source by using the newly defined IVIFS similarity measure. Let $\left\{{E}_{1},{E}_{2},{E}_{3},{E}_{4},{E}_{5},{E}_{6}\right\}$ be the renewable energy sources for electricity generation.

1. $${E}_{1}$$: Tidal energy
2. $${E}_{2}$$: Wind energy
3. $${E}_{3}$$: Solar energy
4. $${E}_{4}$$: Hydropower energy
5. $${E}_{5}$$: Geothermal energy
6. $${E}_{6}$$: Biomass energy

Let $X=\left\{{\epsilon }_{1},{\epsilon }_{2},{\epsilon }_{3},{\epsilon }_{4}\right\}$ be the criterion use to evaluate the efficacy of various renewable energy sources.

1. $${\epsilon }_{1}$$: Availability;
2. $${\epsilon }_{2}$$: Cost;
3. $${\epsilon }_{3}$$_:_ Reliability;
4. $${\epsilon }_{4}$$: Technological Maturity.

**Figure 1:**
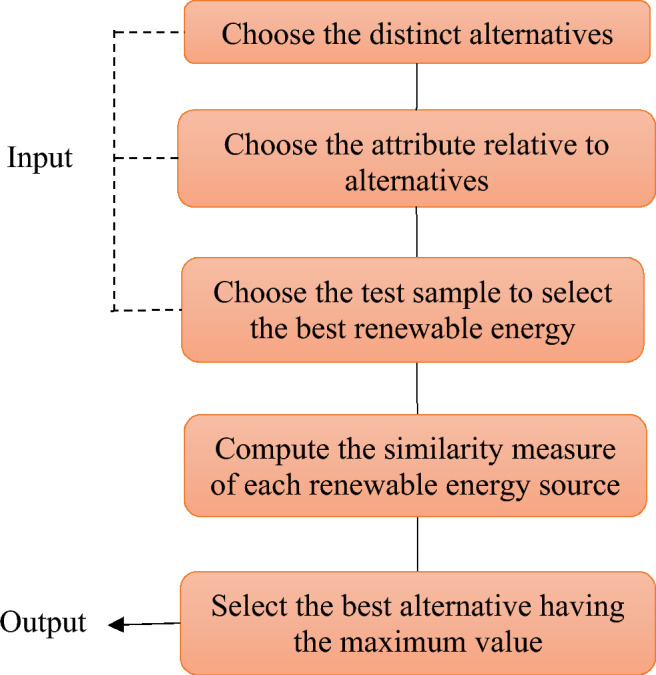
Step-by-step procedure for choosing the best renewable energy source.

A decision making problem using the newly proposed IVIFSs similarity measure is analyzed to assess the six renewable energy sources. Let

$$\Lambda = \left\{\begin{array}{c}\langle {\epsilon }_{1}, \left[0.55, 0.65\right], \left[0.05, 0.10\right]\rangle , \langle {\epsilon }_{2}, \left[0.15, 0.25\right], \left[0.30, 0.35\right]\rangle ,\\ \langle {\epsilon }_{3},\left[0.45, 0.60\right], [0.10, 0.15]\rangle ,\langle {\epsilon }_{4}, \left[0.35, 0.50\right], [0.00, 0.05]\rangle \end{array}\right\}$$

be an IVIFS classified as test sample to evaluate the performance of a specific renewable energy source. The flowchart of the MCDM problem is illustrated in the Figure 1.

In order to address the MCDM problem, we opt for a unique alternative. Next, we identify and consider all the variables that could potentially influence these different choices, and we create a decision matrix. Subsequently, we select a test sample. Once all the input elements have been selected, we proceed to apply the proposed similarity measure to each alternative using the test sample. Ultimately, we choose the alternative with the highest value, which is considered our optimal choice.

***Step 1.***

The information given by decision maker for the above six renewable energy sources are evaluated under IVIF environment and are summarized in Table 2. The normalized matrix is presented in the subsequent Table 3.

**Table 2:** IVIF decision matrix on selecting best renewable energy source.

	${\epsilon }_{1}$	${\epsilon }_{2}$	${\epsilon }_{3}$	${\epsilon }_{4}$
${E}_{1}$	$\left([0.65, 0.75], [0.10, 0.15]\right)$	$\left(\left[0.40, 0.45\right],[0.10, 0.20]\right)$	$(\left[\mathrm{0.65,0.80}\right],\left[0.05, 0.10\right])$	$(\left[0.50, 0.65\right], \left[\mathrm{0.10,0.15}\right])$
${E}_{2}$	$(\left[0.45, 0.55\right], \left[0.00, 0.05\right])$	$(\left[0.50, 0.55\right],\left[0.10, 0.20\right])$	$(\left[0.50, 0.65\right], \left[0.05, 0.10\right])$	$(\left[0.40, 0.55\right], \left[0.05, 0.10\right])$
${E}_{3}$	$(\left[0.35, 0.45\right], \left[0.05, 0.10\right])$	$(\left[0.35, 0.40\right],\left[0.15, 0.25\right])$	$(\left[0.60, 0.75\right], \left[0.10, 0.15\right])$	$(\left[0.65, 0.80\right], \left[0.10, 0.15\right])$
${E}_{4}$	$([0.40, 0.50], [0.00, 0.05])$	$(\left[0.60, 0.65\right],[0.20, 0.30])$	$([0.35, 0.50], [\mathrm{0.00,0.05}])$	$(\left[0.40, 0.55\right], \left[\mathrm{0.00,0.05}\right])$
${E}_{5}$	$\left(\left[\mathrm{0.55,0.65}\right],\left[\mathrm{0.15.0}.20\right]\right)$	$\left(\left[\mathrm{0.35.0}.40\right],\left[\mathrm{0.20,0.30}\right]\right)$	$\left(\left[\mathrm{0.50,0.65}\right],\left[\mathrm{0.05.0}.10\right]\right)$	$\left(\left[\mathrm{0.55,0.70}\right],\left[\mathrm{0.10.0}.15\right]\right)$
${E}_{6}$	$\left(\left[\mathrm{0.50,0.60}\right],\left[\mathrm{0.10.0}.15\right]\right)$	$\left(\left[\mathrm{0.45.0}.50\right],\left[\mathrm{0.15,0.25}\right]\right)$	$\left(\left[\mathrm{0.55,0.70}\right],\left[\mathrm{0.15.0}.20\right]\right)$	$\left(\left[\mathrm{0.45,0.60}\right],\left[\mathrm{0.15.0}.20\right]\right)$

**Table 3:** Normalized IVIF decision matrix.

	${\epsilon }_{1}$	${\epsilon }_{2}$	${\epsilon }_{3}$	${\epsilon }_{4}$
${E}_{1}$	$\left([0.65, 0.75], [0.10, 0.15]\right)$	$\left([0.10, 0.20], [0.40, 0.45]\right)$	$(\left[\mathrm{0.65,0.80}\right],\left[0.05, 0.10\right])$	$(\left[0.50, 0.65\right], \left[\mathrm{0.10,0.15}\right])$
${E}_{2}$	$(\left[0.45, 0.55\right], \left[0.00, 0.05\right])$	$(\left[0.10, 0.20\right], \left[0.50, 0.55\right])$	$(\left[0.50, 0.65\right], \left[0.05, 0.10\right])$	$(\left[0.40, 0.55\right], \left[0.05, 0.10\right])$
${E}_{3}$	$(\left[0.35, 0.45\right], \left[0.05, 0.10\right])$	$(\left[0.15, 0.25\right], \left[0.35, 0.40\right])$	$(\left[0.60, 0.75\right], \left[0.10, 0.15\right])$	$(\left[0.65, 0.80\right], \left[0.10, 0.15\right])$
${E}_{4}$	$([0.40, 0.50], [0.00, 0.05])$	$([0.20, 0.30], [0.60, 0.65])$	$([0.35, 0.50], [\mathrm{0.00,0.05}])$	$(\left[0.40, 0.55\right], \left[\mathrm{0.00,0.05}\right])$
${E}_{5}$	$\left(\left[\mathrm{0.55,0.65}\right],\left[\mathrm{0.15.0}.20\right]\right)$	$\left(\left[\mathrm{0.20,0.30}\right],\left[\mathrm{0.35.0}.40\right]\right)$	$\left(\left[\mathrm{0.50,0.65}\right],\left[\mathrm{0.05.0}.10\right]\right)$	$\left(\left[\mathrm{0.55,0.70}\right],\left[\mathrm{0.10.0}.15\right]\right)$
${E}_{6}$	$\left(\left[\mathrm{0.50,0.60}\right],\left[\mathrm{0.10.0}.15\right]\right)$	$\left(\left[\mathrm{0.15,0.25}\right],\left[\mathrm{0.45.0}.50\right]\right)$	$\left(\left[\mathrm{0.55,0.70}\right],\left[\mathrm{0.15.0}.20\right]\right)$	$\left(\left[\mathrm{0.45,0.60}\right],\left[\mathrm{0.15.0}.20\right]\right)$

***Step 2***. The similarity measure of each alternative ${E}_{i}$ corresponding to the set $\Lambda$ computed by using Definition 4.1.1. and is given by.

$$\mathcal{T}\left({E}_{1},\Lambda \right)=0.752,$$

$$\mathcal{T}\left({E}_{2},\Lambda \right)=0.880,$$

$$\mathcal{T}\left({E}_{3},\Lambda \right)=0.841,$$

$$\mathcal{T}\left({E}_{4},\Lambda \right)=0.989,$$

$$\mathcal{T}\left({E}_{5},\Lambda \right)=0.780,$$

$$\mathcal{T}\left({E}_{6},\Lambda \right)=0.840.$$

***Step 3*** From the above discussion we note that,

$$\mathcal{T}\left({E}_{4} , \Lambda \right)>\mathcal{T}\left({E}_{2} , \Lambda \right)>\mathcal{T}\left({E}_{3} , \Lambda \right)>\mathcal{T}\left({E}_{6} , \Lambda \right)>\mathcal{T}\left({E}_{5},\Lambda \right)>\mathcal{T}\left({E}_{1},\Lambda \right)$$

This means that

$${E}_{4}>{E}_{2}>{E}_{3}>{E}_{6}>{E}_{5}>{E}_{1}$$

Consequently, in view of ranking order, hydropower energy is the best renewable energy source.

### Comparative analysis

In order to ascertain the viability of the suggested similarity metrics, a comparative examination is undertaken utilizing a variety of established methodologies. The outcomes associated with these methodologies are succinctly presented in Table 4.

**Table 4:** Comparative analysis of proposed similarity measure to the existing strategies.

	$S({E}_{1}, B)$	$S({E}_{2}, B)$	$S({E}_{3}, B)$	$S({E}_{4}, B)$	$S\left({E}_{5},B\right)$	$S\left({E}_{6},B\right)$	Ranking
${S}_{1}$	$0.805$	$0.826$	$0.858$	$0.858$	$0.826$	$0.819$	${E}_{4}={E}_{3}>{E}_{2}={E}_{5}>{E}_{6}>{E}_{1}$
${S}_{2}$	$0.765$	$0.800$	$0.812$	$0.826$	$0.800$	$0.787$	${E}_{4}>{E}_{3}>{E}_{2}={E}_{5}>{E}_{6}>{E}_{1}$
${S}_{W}$	$0.729$	$0865$	$0.905$	$0.905$	$0.860$	$0.877$	${E}_{4}={E}_{3}>{E}_{6}>{E}_{2}>{E}_{5}>{E}_{1}$
${S}_{D}$	$0.951$	$0.941$	$0.940$	$0.956$	$0.951$	$0.936$	${E}_{4}>{E}_{5}={E}_{1}>{E}_{2}>{E}_{3}>{E}_{6}$
${S}^{p}$	$0.800$	$0.911$	$0.881$	$0.953$	$0.845$	$0.850$	${E}_{4}>{E}_{2}>{E}_{3}>{E}_{6}>{E}_{5}>{E}_{1}$
$\mathcal{T}$	$0.752$	$0.880$	$0.841$	$0.989$	$0.780$	$0.840$	${E}_{4}>{E}_{2}>{E}_{3}>{E}_{6}>{E}_{5}>{E}_{1}$

From the above table it is quite evident that, the similarity measure ${S}_{1},{S}_{2},{S}_{w}$ and ${S}_{D}$ are unable to rank the alternatives ${E}_{i}$ because.

1. ${S}_{1}\left({E}_{4},\Lambda \right)={S}_{1}\left({E}_{3},\Lambda \right)$ where ${E}_{4}\ne {E}_{3}$ and ${S}_{1}\left({E}_{2},\Lambda \right)={S}_{1}\left({E}_{5},\Lambda \right)$ where ${E}_{2}\ne {E}_{5}$.
2. $${S}_{2}\left({E}_{2},\Lambda \right)={S}_{2}\left({E}_{5},\Lambda \right)$$ where $${E}_{2}\ne {E}_{5}$$.
3. $${S}_{W}\left({E}_{4},\Lambda \right)={S}_{W}\left({E}_{3},\Lambda \right)$$ where $${E}_{4}\ne {E}_{3}$$.
4. $${S}_{D}\left({E}_{5},\Lambda \right)={S}_{D}\left({E}_{1},\Lambda \right)$$ where $${E}_{5}\ne {E}_{1}$$.

However, the ranking of ${S}^{p}$ is same as our proposed similarity measure $\mathcal{T}$. This means that our proposed similarity measure is reasonable and hence can be used to solve MCDM problems.

## Conclusions

Interval valued intuitionistic fuzzy sets (IVIFS) are important for many reasons, such as making it easier to find information, make tough choices, and deal with uncertainty in many fields. Similarity measures are very important in IVIF knowledge because they show how much IVIFS are different or the same. To deal with the fact that information systems are uncertain, different similarity measures have already been set up among IVIFSs. However, the results of most of these measures cannot properly address the MCDM challenges. The present paper has effectively illustrated this aspect by providing numerous examples. To address the shortcomings of existing similarity measures in the IVIF context, a new similarity measure has been proposed in this work. Through a comparison with previously established similarity measures, the current study establishes that the newly defined similarity measure $\mathcal{T}$ is more effective in the IVIF context than the previous measures. In this way, the recently defined similarity measure $\mathcal{T}$ is exceptionally useful for resolving decision-making issues. Additionally, the structural characteristics of the similarity measure $\mathcal{T}$ that has been proposed have been determined. In addition, an algorithm to solve the issues utilizing the recently defined technique has been devised in accordance with IVIF knowledge. In addition, the newly defined similarity metric has been effectively implemented in the selection process for the most optimal renewable energy source aimed at alleviating energy crises. Finally, in accordance with IVIF knowledge, a comparative analysis was performed to establish the validity and suitability of the recently introduced similarity metric $\mathcal{T}$ in relation to the established metrics.

### Limitations of the current study

In the context of MCDM, the method proposed in this work is subject to a number of limitations, despite its enticing advantages. These limitations become apparent when the total score of membership and non-membership is greater than 1 or when neutral membership is involved. These shortcomings can be addressed by using IV Pythagorean and IV picture fuzzy scenarios. The IV Pythagorean and IV picture fuzzy sets have the potential to address the limitations of IVIFS by offering better uncertainty representation, greater aggregation, accessibility, particular modifications, and more rigorous mathematical theory. Furthermore, our proposed approach primarily addresses one-dimensional issues. To handle scenarios involving 2-D information about a physical phenomenon, the utilization of the complex IVIF approach becomes an effective option.

### Future goals of the current study

One of our primary aims will be to rectify the limitations of the current study by introducing the proposed similarity measure in the framework of IV Pythagorean and IV picture fuzzy sets in our future study. In addition, the concept of complex IVIFS will be utilized to enhance the validity of the proposed similarity measure in solving MCDM problems with 2-dimensional information. Moreover, our focus will also be to implement proposed similarity measures to address MCDM problems across various physical phenomena, including environmental protection, clustering, and medical diagnosis using IVIF information.

## Human and animal rights

The authors confirm that their study does not involve any humans/study participants or subjects/patients.

## Data Availability

All data generated or analyzed during this study are included in this article.
